# Sensory initiation of a co‐ordinated motor response: synaptic excitation underlying simple decision‐making

**DOI:** 10.1113/JP270792

**Published:** 2015-07-30

**Authors:** Edgar Buhl, Stephen R. Soffe, Alan Roberts

**Affiliations:** ^1^ School of Biological SciencesUniversity of BristolBristolUK

## Abstract

**Key points:**

- Deciding whether or how to initiate a motor response to a stimulus can be surprisingly slow and the underlying processes are not well understood.
- The neuronal circuitry that allows frog tadpoles to swim in response to touch is well characterized and includes excitatory reticulospinal neurons that drive swim circuit neurons.
- Build‐up of excitation to reticulospinal neurons is the key decision‐making step for swimming. Asymmetry in this build‐up between the two sides allows bilateral initiation at the same time as avoiding inappropriate co‐activation of motor antagonists.
- Following stronger stimuli, reticulospinal neurons are excited through a trigeminal nucleus pathway and swimming starts first on the stimulated side. If this pathway fails or is lesioned, swimming starts later on the unstimulated side.
- The mechanisms underlying initiation of a simple tadpole motor response may share similarities with more complex decisions in other animals, including humans.

**Abstract:**

Animals take time to make co‐ordinated motor responses to a stimulus. How can sensory input initiate organized movements, activating all necessary elements at the same time as avoiding inappropriate co‐excitation of antagonistic muscles? In vertebrates, this process usually results in the activation of reticulospinal pathways. Young *Xenopus* tadpoles can respond to head‐skin touch by swimming, which may start on either side. We investigate how motor networks in the brain are organized, and whether asymmetries in touch sensory pathways avoid co‐activation of antagonists at the same time as producing co‐ordinated movements. We record from key reticulospinal neurons in the network controlling swimming. When the head skin is stimulated unilaterally, excitation builds up slowly and asymmetrically in these neurons such that those on both sides do not fire synchronously. This build‐up of excitation to threshold is the key decision‐making step and determines whether swimming will start, as well as on which side. In response to stronger stimuli, the stimulated side tends to ‘win’ because excitation from a shorter, trigeminal nucleus pathway becomes reliable and can initiate swimming earlier on the stimulated side. When this pathway fails or is lesioned, swimming starts later and on the unstimulated side. Stochasticity in the trigeminal nucleus pathway allows unpredictable turning behaviour to weaker stimuli, conferring potential survival benefits. We locate the longer, commissural sensory pathway carrying excitation to the unstimulated side and record from its neurons. These neurons fire to head‐skin stimuli but excite reticulospinal neurons indirectly. We propose that asymmetries in the sensory pathways exciting brainstem reticulospinal neurons ensure alternating and co‐ordinated swimming activity from the start.

**Abbreviations**
dlc: dorsolateral commissural neuron
dIN: descending interneuron
IQR: interquartile range
rdlc: rostral dorsolateral commissural neuron
tIN: trigeminal descending interneuron
tSt: trigeminal sensory touch receptor

## Introduction

There has been extensive study on how humans and other animals initiate directed movements such as eye saccades, reaching and locomotion in response to sensory stimulation (Gold and Shadlen, 2007; Dubuc *et al*. 2008; Grillner *et al*. 2008; Jordan *et al*. 2008; Goulding, 2009; Romo and de Lafuente, 2013). An emerging consensus is that the decision process to initiate co‐ordinated movements is complex and much slower than simpler reflex responses or the ballistic escape responses of invertebrates or fish (crayfish: Olson and Krasne, 1981; fish: Korn and Faber, 2005). Sensory information is considered to travel to higher brain centres. Commands are then sent to motor centres in the brainstem to activate eye or limb movements, or locomotion. This decision process is interactive and takes time (often > 100 ms) involving the simultaneous activity of large numbers of neurons in different brain regions (Romo and de Lafuente, 2013). In vertebrates, a critical step is the activation of reticulospinal neurons in the brainstem, which then activate motoneurons to produce movements (Dubuc *et al*. 2008; Jordan *et al*. 2008; Baker, 2011). Unfortunately, the detailed neuronal pathways activating reticulospinal neurons during a decision are still unclear and major questions remain. For example, what happens during the decision process to ensure that reticulospinal neurons act together to initiate movement? How is the direction of the first movement determined? How is activation of antagonistic muscles ensured at the same time as co‐activation is avoided?

To understand the decision to initiate co‐ordinated movement at the neuronal level, we need a simpler model for investigation. Larval zebrafish have provided valuable insights into neurons controlling swimming (Kimura *et al*. 2013), but our choice is the hatchling *Xenopus* tadpole, which will swim in response to touch on one side of the head (Boothby and Roberts, 1995). If the touch is sufficiently strong, the tadpole flexes to either side and then swims off. In immobilized tadpoles, we can use skin stimulation to initiate fictive swimming recorded in ventral roots and define the neurons and pathways controlling locomotion. Recent studies have shown that a small population of electrically coupled reticulospinal neurons drive swimming on a cycle‐by‐cycle basis. These descending interneurons (dINs) have been characterized anatomically and physiologically. They form a longitudinal column extending from the brainstem into the spinal cord and fire once on each cycle of swimming to drive similar firing in all other neurons active during swimming, including motoneurons (Soffe *et al*. 2009; Roberts *et al*. 2010; Li, 2011; Moult *et al*. 2013). We also have detailed information on the head‐skin sensory system. On each side, 50–80 trigeminal sensory touch (tSt) receptors innervate the head skin with ‘free’ nerve endings (Roberts, 1980; Hayes and Roberts, 1983). Each one fires once to touch and projects a single axon through the hindbrain. Their excitation converges onto a hindbrain nucleus of ∼20 trigeminal nucleus neurons (tINs) (Buhl *et al*. 2012). The tINs directly excite reticulospinal dINs and, if this excitation following head‐skin stimulation is sufficient, the whole electrically coupled dIN population is recruited and swimming starts. Modelling of the dIN population response to sensory input has shown that electrical coupling between dINs is critical to this all‐or‐none pattern of recruitment when swimming starts (Hull *et al*. 2015). Our work has defined how stimulation of trigeminal head‐skin afferents can activate a trigeminal nucleus and the reticulospinal dINs to initiate swimming on the stimulated side of the body. However, a significant problem remains. The initiation of swimming requires both sides to be excited following a stimulus to one side and so, as in the spinal cord (Li *et al*. 2003), there must be sensory pathway neurons with commissural projections to carry excitation to the unstimulated side.

When animals decide to make even a simple movement in response to a sensory stimulus, the activation of different antagonistic muscles must be co‐ordinated. This is true for eye movements, reaching movements or the initiation of locomotion. In fish and tadpoles, there are basically only two segmented blocks of trunk swimming muscles, one on each side, and so co‐ordination should be simpler. When skin touch initiates swimming, motoneuron firing is driven by reticulospinal neurons, but how are these neurons excited on both sides of the body while avoiding synchronous co‐activation? Once established, swimming contractions alternate on opposite sides; when reticulospinal neurons on both sides are first excited, their firing must be co‐ordinated such that co‐activation, and therefore synchronous contraction of muscles on both sides, is avoided. This is not trivial: synchronous firing is a stable state in simple rhythmic model networks coupled by reciprocal inhibition (Wang and Rinzel, 1992) and can occur transiently in models of the tadpole swimming network (Roberts *et al*. 2014). Curiously, it has occasionally been seen in whole‐cell recordings of tadpole reticulospinal neurons following skin stimulation (Li *et al*. 2014). Because synchronous bilateral motor activity appears to make no behavioural sense for the tadpole, what prevents it during initiation?

To resolve questions about the role of reticulospinal neurons in the initiation of co‐ordinated swimming, we examine *Xenopus* tadpole behavioural responses to head‐skin stimulation. We then use immobilized tadpoles first to record ventral root responses to head‐skin stimuli, and then to make whole‐cell recordings from hindbrain reticulospinal neurons controlling swimming. Video and ventral root recordings show that, when the head is touched on one side, the first flexion of swimming can be on either side, although this occurs earlier if swimming starts on the stimulated side than if it starts on the unstimulated side. Using paired whole‐cell recordings from the reticulospinal neurons driving swimming, we then demonstrate that the sensory pathways to the two sides differ and lead to an organized but asymmetrical build‐up of excitation. As a result, alternating firing of the reticulospinal neuron populations on the two sides is established right from the start and synchronous activity is avoided. Lesions are used to locate the commissural pathway neurons activated by sensory stimulation and we then record their responses. Whole‐cell recordings and lesions show that sidedness is determined by the success or failure of the stimulated‐side pathway. To threshold stimuli, this pathway can fail and the tadpole responds unpredictably. However, to stronger stimuli, when this pathway is reliable, the tadpole responds quickly and flexes to the stimulated side first. We conclude that asymmetry between skin sensory pathways on the two sides allows the bilateral initiation underlying a decision to swim at the same time as preventing unwanted co‐activation of antagonists.

## Methods

### Animals

Procedures for obtaining developmental stage 37/38 (Nieuwkoop and Faber, 1956) hatchling *Xenopus laevis* (Daudin) tadpoles comply with UK Home Office regulations. All unregulated experiments on the tadpoles have been approved after local ethical committee review. All chemicals were obtained from Sigma (Poole, UK). Experiments were performed at 18–22°C.

### Behaviour

The response of the tadpoles to a touch stimulus (hair, 10 μm tip) applied to the head was analysed using high‐speed video recording (Exilim EX‐F1; Casio, Tokyo, Japan) with 300 frames s^–1^. For this, the animals were placed in a small Petri dish (diameter 8 cm) base‐filled on one side with a layer of Sylgard (Dow Corning, Midland, MI, USA) in an upright (dorsoventral) position within a groove cut into the edge of the Sylgard so that the head and cement gland were unrestrained. An array of LED lights provided even illumination from below. The videos were cut and analysed using ImageReady video editing software (Adobe Systems Inc., San Jose, CA, USA) and ImageJ image processing software (NIH, Bethesda, MD, USA).

### Electrophysiology

Following brief anaesthesia (in 0.1% MS‐222 3‐aminobenzoic acid ester), tadpoles were immobilized using 10 μm α‐bungarotoxin in saline (115 mm NaCl, 3 mm KCl, 2 mm CaCl_2_, 2.4 mm NaHCO_3_, 1 mm MgCl_2_, 10 mm Hepes adjusted to pH 7.4 with NaOH) for 20–30 min. For experiments involving only ventral root recordings of fictive swimming, the animals were then pinned to a rotatable Sylgard‐coated platform in a small (∼2 ml) recording chamber. Skin was removed from either side of the trunk to allow fire polished glass suction electrodes, with tip openings of 40–60 μm and filled with saline, to be placed at clefts between myotomes where motoneuron axons run. Ventral root signals were amplified by a differential amplifier (gain: 1000; SOBS, University of Bristol, Bristol, UK) and filtered (low: 30 Hz, high: 1 kHz). Fictive swimming was initiated by stimulation of the head skin with either a touch stimulus or with a brief (0.1 ms) current pulse delivered using a glass suction electrode, again with a tip opening of 40–60 μm filled with saline. This excites the peripheral processes of sensory neurons that enter the brain via the trigeminal nerve (Roberts, 1980).

Lesions were performed by hand under a M205C stereo microscope (Leica, Wetzlar, Germany) using mounted pins made from 50 μm diameter tungsten wire, etched to a fine point. The position of lesions was judged by eye relative to clear landmarks: otic capsule, hindbrain segments (rhombomeres) and myotome segments. To remove the tIN population unilaterally, superficial internal tissue was removed on one side of the hindbrain in rhombomeres 2 to 4 from the mid‐line to the edge enclosing the whole extent of the tIN population (Buhl *et al*. 2012). Care was taken not to damage the underlying marginal zone including the axons of trigeminal sensory neurons. The extent of the lesion was judged by prior extensive experience of tIN recordings and effects on swim initiation.

For experiments involving whole‐cell recording, immobilized animals were pinned to a rotatable Sylgard‐coated platform, and the roof of the hindbrain and parts of the inner surface removed to reveal neuron somata. The animals were then repinned in a small recording chamber (∼1 ml) where neuron somata could be seen using a 40× water immersion lens on an upright E600FN microscope (Nikon, Tokyo, Japan). Once again, fictive swimming was initiated by stimulation of the head skin with a brief (0.1 ms) current pulse and recorded from the ventral roots using saline‐filled suction electrodes applied to intermyotome clefts. Whole‐cell current clamp recordings were made with an Axoclamp 2B (Axon Instruments Inc., Foster City, CA, USA) in bridge mode, filtered (at 30 kHz) and digitized (sampling rate: 10 or 20 kHz, ADC resolution: 16 bit) using a CED Power1401mkII interface (Cambridge Electronic Design Ltd, Cambridge, UK) and standard software Signal 3 and 4 (Cambridge Electronic Design Ltd). Patch pipettes were filled with 0.1% neurobiotin in intracellular solution (100 mm K‐gluconate, 2 mm MgCl_2_, 10 mm EGTA, 10 mm Hepes, 3 mm Na_2_ATP, 0.5 mm NaGTP adjusted to pH 7.3 with KOH) and had resistances in the range of 5–15 mΩ. The liquid junction potential of this solution was measured as +11 mV but, for better comparison with previous data, the values have not been corrected for this.

### Electroporation

To identify and record from neurons activating the dINs, animals were dissected as before and parts of the hindbrain were removed to access dINs. A glass electrode (2–3 MΩ) was filled with the fluorescent marker AlexaFluor488 (dextran, 10000 MW; Invitrogen, Carlsbad, CA, USA) and placed at the presumed position of the dIN dendrites. To label neurons projecting onto these dendrites, a positive current pulse train (10 ms, 50 Hz, 20 V) was applied for 15–30 min and the electrode position was altered occasionally during the electroporation process. After 2–3 h, the labelled neurons could be seen under a fluorescence microscope and were then recorded together with a dIN.

### Anatomy

The anatomical identity of the recorded neurons was routinely checked after the experiments using a standard avidin‐biotin technique with diaminobenzidine as chromogen. After the experiments, the animals were left in the saline for 20–25 min, allowing neurobiotin to diffuse throughout the whole cell. The animals were fixed in 2% glutaraldehyde for at least 2 h, washed in PBS (120 mm NaCl in 0.1 m phosphate buffer) and again in PBS containing 1% TritonX before labelling with ExtrAvidin (dilution 1:200) for 3 h. The animals were then washed again in 0.1 m PBS, the neurons stained in 0.8% 3,3'‐diaminobenzidine in phosphate buffer, and then again in buffer containing 3,3'‐diaminobenzidine and 0.03% H_2_O_2_ for 5 min each. After washing in tap water, the brain and spinal cord were exposed and the specimen dehydrated in an ascending alcohol series and cleared in methyl benzoate. Specimens were mounted ventral side down between coverslips with DePeX on a reversible aluminium microscope slide. Once mounted, the hindbrain lay open along the ventral mid‐line analagous to an open book. Neurons were observed using a 100× oil immersion lens and traced using a drawing tube. Photographs of the brain were obtained using a 20× objective lens and a CCD camera (DP 200; DeltaPix, Maalov, Denmark) and arranged in Photoshop (Adobe Systems Inc., San Jose, CA, USA). Measurements were made from the scale drawings and corrected for shrinkage during processing (1.28×). The position of the recorded cell bodies was measured during recording using a micrometre connected to the microscope. It is possible that cell bodies could move and change shape slightly during recording and processing.

### Statistical analysis

Data analysis was performed using purpose‐written routines for Minitab (Minitab Ltd, Coventry, UK). The latencies of EPSPs or the first action potential were measured as the time between the stimulus and the onset of the postsynaptic potential and averaged for each cell. Additionally, for EPSPs, the amplitude, the 10–90% rise time, the time to peak, the slope and the width at half‐peak‐amplitude were measured. The recorded traces, photographs and drawings were imported into Corel Draw (Corel Corp., Ottawa, Canada) for final layout. The statistical tests used are as stated throughout the Results and were performed using Minitab (Minitab Inc., State College, PA, USA) or Prism (GraphPad Software Inc., La Jolla, CA, USA). *P* < 0.05 was considered statistically significant. If not stated otherwise, measurements are expressed as the mean ± SD or the median and interquartile range (IQR).

## Results

### Head touch initiates swimming behaviour

**Figure 1:**
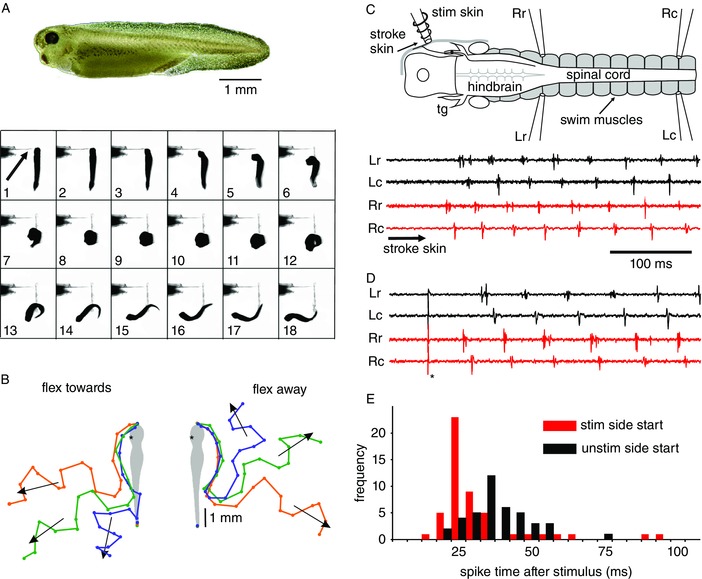
**Swimming in response to head‐skin stimulation** *A*, tadpole and video frames (150 frames s^–1^) showing a tadpole viewed from above responding to touch on the left side of the head with a fine hair (arrow) by flexing to the left (frames 4–10) and then swimming off (frames 14–18). *B*, tracings from six similar videos after head stimulation on the left side (asterisk) plot head position to show flexion to left or right and swimming in a range of directions (arrows). *C* and *D*, an immobilized tadpole showing the brain, spinal cord and innervation of head skin by a sensory neuron in the trigeminal ganglion (tg). The positions of suction electrodes to record ventral root activity from swimming muscles (Rr, Rc, Lr, Lc) and to stimulate the right head skin are indicated. Fictive swimming can start on the stimulated side after touch (arrow, *C*) or current pulse (asterisk, *D*) stimulation to the head skin. *E*, histogram of latencies to the start of swimming in immobilized tadpoles following skin stimulation on the same (red bars) or opposite (black bars) side of the head. Bin size = 5 ms.

When a tadpole, lying on the bottom of a dish with its cement gland detached from the substrate, is touched anywhere on the head using a fine hair (10 μm tip), high‐speed videos show that it flexes its trunk and then starts swimming, bending alternately to right and left at 10–20 Hz (Fig. 1 *A*) (Roberts, 1990). Following gentle manually controlled touch, the initial flexion is strong and can be towards (36.7%) or away from the stimulated side (*n* = 30 tadpoles). The animal then swims away in an unpredictable direction (Fig. 1 *B*) (Boothby and Roberts, 1995).

Responses to head‐skin stimulation can also be studied in immobilized tadpoles by recording motor nerve activity in the ventral root with suction electrodes applied between swim muscles in the trunk (Fig. 1 *C*). A touch or stroke with a fine hair or a 0.1 ms current pulse applied via a suction electrode to the head skin can evoke fictive swimming (Fig. 1 *C* and *D*). As with the behaviour, the first motor response to stimulating the head skin can be on either side of the animal (stimulated side: 49%, *n* = 10 tadpoles and 95 initiations). Following only suprathreshold electrical skin stimulation, reaction times measured to the start of fictive swimming were significantly shorter on the stimulated side (range 15–87 ms, median 25 ms, *n* = 50 episodes) than on the unstimulated side (range 20–71 ms, median 35 ms, *n* = 41 episodes; generalized linear model on 1/latency: *P* = 0.01) (Fig. 1 *E*). Stimulation of the head skin therefore leads to swimming activity that can initiate on either side, although there is an asymmetry because swimming generally starts earlier on the stimulated side than on the unstimulated side.

### Recruitment of swim reticulospinal neurons on both sides

To study the pathways responsible for asymmetries in the initiation of the swimming behaviour, we recorded from key neurons in the swim network. Reticulospinal and spinal dINs are electrically coupled, excite each other, fire a single action potential on each swim cycle, fire first on each cycle to excite all other swim neuron types on the same side, show post‐inhibitory rebound firing to brief negative current pulses when they are held depolarized, and show pacemaker firing during NMDA receptor activation (Roberts *et al*. 2010; Li, 2011; Moult *et al*. 2013). We have recently defined a two‐synapse pathway from head‐skin trigeminal touch afferents via tINs to ipsilateral dINs (Buhl *et al*. 2012). The same study also showed that dIN firing precedes ventral root activity when head‐skin stimulation initiates swimming starting on the stimulated side. Because swimming can start on the stimulated or unstimulated side, we therefore expect that dINs on both sides will receive excitation.

**Figure 2:**
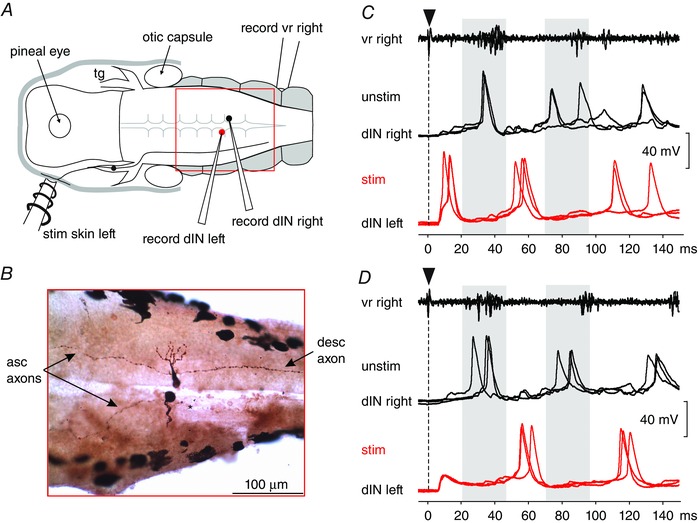
**Excitation and alternating firing of reticulospinal dINs on both sides after head‐skin stimulation** *A*, positions of recording and stimulating electrodes. *B*, photograph of a recorded pair of dINs on either side of the hindbrain (dorsal view, rostral to the left; red rectangle in *A*) with soma, dendrites and both ascending and descending axons. The descending axon of the left side dIN was damaged during the dissection (at*). *C* and *D*, each panel shows three overlain responses from the right ventral root and the pair of dINs shown in *B*. In each case, swimming starts following a left head‐skin stimulus (at arrowhead) and grey bars show the phase when spikes occur on the unstimulated side. *C*, the first dIN spike is at short latency on the left, stimulated side (red). *D*, there is a short‐latency EPSP in the left dINs but the first spike is later on the unstimulated right side (black) and is followed by the first spike on the stimulated side.

**Figure 3:**
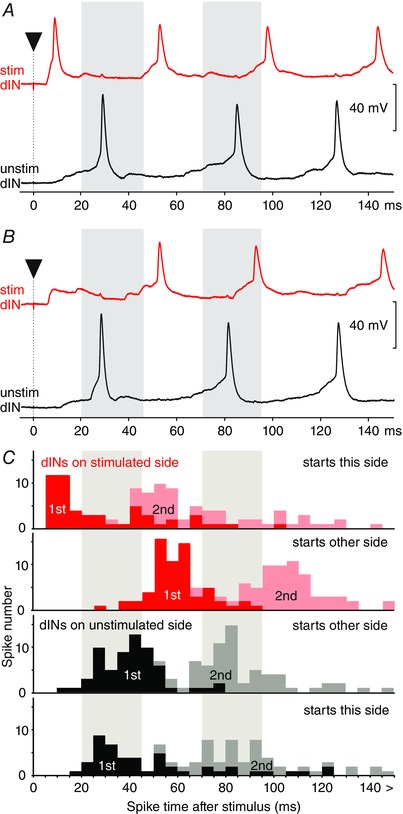
**Alternating pattern of first and second reticulospinal dIN spike times on each side of the body following head‐skin stimulation** *A* and *B*, responses from a pair of dINs different from the one shown in Fig. 2. In each case, activity starts following a head‐skin stimulus (at arrowhead) and grey bars show the phase when spikes occur on the unstimulated side. *A*, the first dIN spike is at short latency on the stimulated side (red), whereas, in *B*, the first spike is later on the unstimulated side (black) and each is followed by the first spike on the other side. *C*, plots show spike occurrences on each side at different times after a stimulus to one side of the head (red and pink bars are the stimulated side; black and grey are the unstimulated side). Measures are from 230 responses in six dIN pairs. Bin size = 5 ms.

To identify asymmetries between the responses of dINs to head‐skin stimulation on different sides of the body, we made whole‐cell recordings from pairs of reticulospinal dINs on opposite sides (*n* = 6 pairs) (Fig. 2). These data were supplemented by recordings from single dINs on each side (*n* = 50). All dINs were identified by their responses to injected current, activity during swimming and anatomy following neurobiotin injection (Li *et al*. 2006). In response to a weak electrical stimulus to the head skin, dINs on both sides received excitation. To stronger stimuli, this excitation summed and dIN firing occurred. In 95.4% of cases, dIN firing preceded the first ventral root burst of swimming (219 swim initiations in six pairs of dINs) (Fig. 2 *C* and *D*) consistent with the conclusion that recruitment of the dIN population initiates swimming (Buhl *et al*. 2012). The initial excitation on the stimulated side rose rapidly to a peak that could lead to firing, whereas excitation on the unstimulated side was smaller but rose steadily to evoke later firing (Figs 2 *C* and *D* and 3 *A* and *B*) (for analysis, see below). It was surprising that, when the dIN on one side fired, there was often no obvious sign of reciprocal inhibition in the dIN on the other side.

The patterns of dIN spike firing times on the two sides were very different, with firing alternating on the two sides (measures are from 230 responses in six dIN pairs). On the unstimulated side (Fig. 3 *C*; black bars), the first dIN spikes had a similar and broadly unimodal distribution, regardless of whether ventral root firing on this side started first (median delay of 39 ms; IQR 29–44 ms) or second (median delay of 33 ms; IQR 29–42 ms). These spike times were not significantly different (Mann–Whitney test: *P* = 0.22). On the stimulated side, however (Fig. 3 *C*; red bars), spike delays had two broad peaks. The first peak corresponds to the cases when ventral root firing started first on the stimulated side (median delay of 12 ms; IQR 10–17 ms) and spikes occur on the early peak of excitation (Fig. 3 *A*; red traces). If ventral root firing started first on the unstimulated side (at ∼30–45 ms), then there is a later peak of spikes on the stimulated side (Fig. 3 *B*; red traces) with a median delay of 57 ms (IQR 52–62 ms). The timing of these later spikes is the same as the timing of the second burst of spikes (i.e. next cycle) on the stimulated side when this side started first (55 ms; IQR 49–63 ms; Mann–Whitney test: *P* = 0.67). The longer delays to dIN spikes on the unstimulated side correspond to the longer delays to the start of ventral root activity at the onset of swimming described above (Fig. 1 *E*; black bars). Repeated stimulation with a stimulus intensity just above the threshold to evoke swimming showed that the first dIN to fire can be on either side (46% stimulated side; *n* = 117 stimulations in 10 dINs). Swimming starts on the side where the recorded dIN fires first.

Measurements of the firing times of reticulospinal dINs after head‐skin stimuli show that they fall into distinct timing windows, which ensures that neurons on the stimulated and unstimulated sides do not fire at the same time but in alternation from the start of swimming. This dIN firing appears to result from asymmetrical synaptic drive, which ensures that dINs on the two sides fire after ‘preferred’ delays. Although the delay on the unstimulated side appears to be fixed (Fig. 3; black bars), the stimulated side fires either earlier or later than the unstimulated side (Fig. 3; red bars). Aiming to explain these differences, we examined the synaptic excitation to dINs on each side following head‐skin stimulation.

### Synaptic excitation underlying reticulospinal neuron recruitment

**Figure 4:**
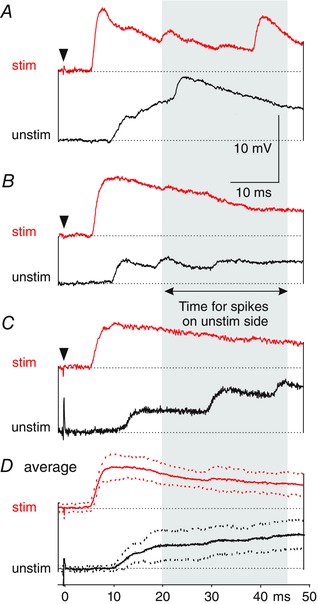
**Asymmetry in EPSP responses to low‐level skin stimulation in dINs on stimulated (red) and unstimulated (black) sides** *A*–*C*, example EPSPs in response to low level stimulation show differences between the two sides in recordings from three different dIN pairs. *D*, averages of 10 responses from records in *C* (continuous lines) show that the stimulated side EPSPs are earlier and rise faster. Dotted lines either side of curves are the SDs.

To investigate the excitatory synaptic drive to the reticulospinal dINs on each side, we measured their responses to the skin stimulus level subthreshold for swim initiation in paired and single recordings. The compound EPSPs seen in dINs differed on the two sides (Fig. 4) and were analysed for 10 EPSPs in 10 dINs for each side. The delays to EPSPs on the stimulated side were slightly shorter (7.0 ± 1.7 ms) than those on the unstimulated side (9.6 ± 1.8 ms; Mann–Whitney test: *P* < 0.0001). However, the main difference was that EPSPs on the stimulated side were faster rising than contralateral EPSPs (10–90% amplitude in 2.2 ± 0.6 ms, slope: 3.1 ± 1.2 mV ms^–1^ compared to 16.7 ± 6.9 ms and 0.5 ± 0.5 mV ms^–1^; both *t*‐test: *P* < 0.0001) (Fig. 4 *D*). EPSPs on the stimulated side, which we assume are mediated by direct synapses from trigeminal nucleus tINs (Buhl *et al*. 2012), fell away after an early peak. EPSPs on the unstimulated side, mediated by a presently unidentified pathway that we assume must fire repetitively, kept increasing. The sensory‐evoked EPSPs in dIN reticulospinal neurons on each side of the body are therefore asymmetric, possibly explaining the differences in first‐spike timings (Figs 2 *C* and *D* and 3).

### Effects of lesions to reticulospinal neuron sensory input pathway

**Figure 5:**
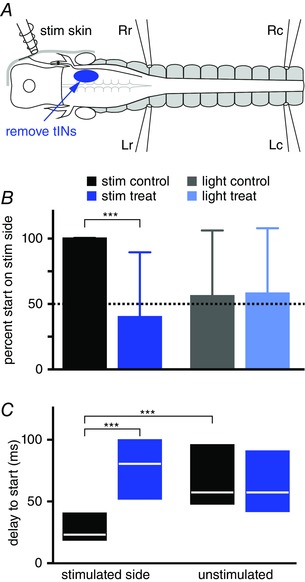
**Effects of removing the tIN population on one side on swimming responses to head‐skin stimulation** *A*, stimulation and recording set‐up and area where tINs were removed (blue). *B*, side of first ventral root response (mean ± SD) to electrical stimulation and light dimming in control (black) and treated animals with tINs removed on the stimulated side (blue). Responses to light were similar before and after treatment. *C*, delays to first ventral root response (median, IQR) on the stimulated and unstimulated side following head‐skin stimulation. Delays increase significantly after removal of the tINs on the treated side only. Asterisks indicate a significant difference at *P* < 0.0001 (Mann–Whitney test).

Behavioural experiments show that swimming can start on either side following stimulation of the head skin on one side (Fig. 1) and our recordings show that, in addition to slowly rising excitation, the reticulospinal dINs on the stimulated side receive short‐latency, fast‐rising excitation from tINs (Buhl *et al*. 2012). In response to stronger stimuli, these tINs fire more, with a greater probability of activating dINs. Our hypothesis is that, if the tIN excitation causes early, same‐side dIN spiking, swimming starts on the stimulated side, whereas, if tIN excitation does not cause early spiking, swimming starts on the unstimulated side. To test whether the ipsilaterally projecting tIN population could influence the side on which swimming starts, we surgically removed the region of hindbrain containing the defined cluster of tIN somata on one side at the same time as leaving the trigeminal central axon projections intact (Fig. 5 *A*). Ventral root recordings from operated animals (*n* = 5; 10 initiations each) showed that, following lesioning, swimming could still be initiated in a well‐organized pattern from the start without any synchronous ventral root activity on both sides (not shown).

In response to strong stimuli (twice threshold to initiate swimming), control animal swimming started reliably on the stimulated side (100%) (Fig. 5 *B*) and removal of the tIN population led to significantly fewer starts on the stimulated side (40%; Mann–Whitney test: *P* < 0.0001). As a control, swimming was initiated by dimming the light (Roberts, 1978) and responsiveness and sidedness was similar before (56% right side) and after tIN removal (58% right side; Mann–Whitney test: *P* = 0.84). The delay to the first ventral root burst on the stimulated side of 23 ms (median, IQR 19–40 ms) was significantly shorter than on the unstimulated side (57 ms, IQR 48–95 ms, Mann–Whitney test: *P* < 0.0001) (Fig. 5 *C*). Removal of the tINs increased this delay only on the stimulated side to 80 ms (IQR 52–99 ms, Mann–Whitney test: *P* < 0.0001) at the same time as leaving the unstimulated, intact, control side unaffected (treated: 57 ms, IQR 42–90 ms, Mann–Whitney test: *P* = 0.86).

Taken together, these results suggest that the population of the tINs, forming the trigeminal nucleus, is responsible for the short‐latency ventral root response on the stimulated side. The source of the slower rising excitation following head‐skin stimulation and seen in dINs on each side of the body is evaluated in the Discussion. Even after tIN lesioning, no synchronous left–right motor activity was seen at the start of swimming in any recordings. This means that, in response to a stimulus to the head‐skin, the populations of dINs on each side receive asymmetrical input independent of, and in addition to, the asymmetric input from tINs. We propose that this asymmetrical input to dINs leads to reliable alternating motor activity right from the start of swimming.

### Lesion experiments locate commissural sensory pathway in hindbrain

**Figure 6:**
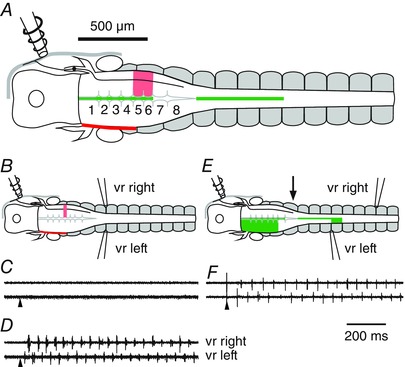
**Lesion experiments showing where trigeminal excitation can cross to the unstimulated side in the caudal hindbrain** *A*, summary of regions of the hindbrain where unilateral transection prevented swimming (pink) or where mid‐line cuts (green) still allowed the initiation of bilateral swimming following a head‐skin stimulus applied to the right side (rhombomeres numbered). The trigeminal ganglia on the left side were severed to prevent contralateral sensory access (red line). *B*, example of a rostral unilateral transection (pink line). *C*, ventral root recordings show this lesion prevented swim initiation to a head‐skin stimulus (arrowhead) but not to a trunk‐skin stimulus (*D*). *E*, example of a mid‐line lesion (green line) that only left a short intact region of caudal hindbrain (arrow). *F*, this short intact region was sufficient to allow initiation of bilateral swimming following a head‐skin stimulus to the right side. Ventral root recording positions on right (upper traces) and left (lower traces) sides are indicated in *B* and *E*.

To attempt to explain the basis for asymmetry in the sensory excitation to reticulospinal dINs following head‐skin stimulation, the next step was to determine the pathway crossing to dINs on the unstimulated side. Trigeminal afferents, tINs and dINs all exclusively project ipsilaterally. We therefore used lesions with electrical stimulation of the head‐skin on the right side to localize areas where commissural axons could carry excitation to the unstimulated (left) side and allow swimming (*n* = 16 tadpoles) (Fig. 6 *A*). Unilateral transection of the right‐side hindbrain anywhere in rhombomeres 5 and 6 (Fig. 6 *A*; pink) blocked the initiation of swimming (*n* = 5) (Fig. 6 *B* and *C*), although swimming could still be evoked by stimulating the trunk skin (Fig. 6 *D*). These lesions suggest that there was no sufficient commissural pathway in the rostral part of the hindbrain. However, mid‐line lesions that cut most commissural connections between the two sides of the hindbrain and rostral spinal cord, but left some intact caudally in rhombomeres 7 to 8, did not block the initiation of swimming (*n* = 11) (Fig. 6 *E* and *F*). These experiments suggest that sensory excitation from the trigeminal afferent neurons innervating the head skin crosses to the other side in the caudal hindbrain at the level of rhombomeres 7 and 8. It should be noted that, although axons crossing at this level are sufficient to allow initiation of swimming, suitable crossing axons are not restricted to this region: mid‐line cuts that included rhombomeres 7 and 8 (*n* = 12) did not block initiation unless extended rostrally and/or caudally. Because the aim of the present study was to localize a region for further investigation, we did not attempt to define the extent of additional crossing.

### Head‐skin stimuli recruit commissural sensory pathway neurons

In the spinal cord, a dorsolaterally positioned longitudinal column of sensory projection neurons with commissural axons is strongly and monosynaptically excited by trunk skin sensory Rohon‐Beard neurons and the neurons fire at high frequency during above‐threshold current injection. These dorsolateral commissural neurons (dlcs) project to the opposite side where they make glutamatergic synaptic connections (Clarke and Roberts, 1984; Roberts and Sillar, 1990; Li *et al*. 2003). If the most rostral members of the dlc population lying in the hindbrain were excited by the descending axons of trigeminal afferents, they could carry excitation from head‐skin touch to the opposite side. We therefore searched for such neurons by making whole‐cell recordings with neurobiotin filling from dorsolaterally located somata in the caudal hindbrain. To identify these neurons for recording, we backfilled their crossing axons by electroporating the fluorescent dye AlexaFluor488 using an electrode in the marginal zone on the opposite side.

**Figure 7:**
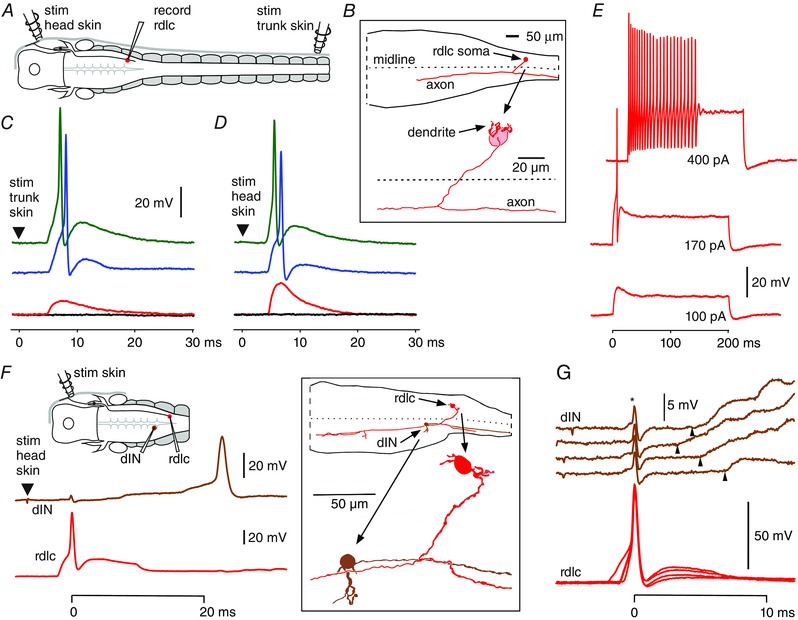
**Sensory pathway rostral dorsolateral commissural neurons (rdlcs) respond to head and trunk skin stimulation** *A*, dorsal view showing the position of the stimulating and recording electrodes. *B*, rdlc neurobiotin filling showing the extent of the axon in hindbrain, with enlarged tracing of rdlc with soma, dendrites and axon crossing the mid‐line and ascending and descending on the contralateral side. *C* and *D*, traces showing rdlc response to ipsilateral trunk and head‐skin stimulation (arrowhead) of increasing strength from black = no response, red = EPSP alone, blue = spike, green = response to strong stimulus. *E*, current injected into an rdlc evokes a single spike at threshold and fast multiple firing at higher levels. *F*, showing the position of the electrodes for paired recording and a record of responses of rdlc and dIN to right head stimulus that starts swimming, as well as low and higher magnification tracings of the recorded cells in the box. *G*, responses of another rdlc/dIN pair to head‐skin stimuli, aligned by rdlc spikes to show EPSPs with variable delays (arrowheads) and shapes. Coupling artefact indicated by an asterisk (*).

We named those neurons in the hindbrain responding at short latency to trunk and head‐skin stimulation, and with evidence of commissural axon projections, rostral dorsolateral commissural neurons (rdlcs). These neurons (*n* = 14) lay in a region 560–880 μm from the mid‐hindbrain border. Similar to dlcs in the spinal cord, they were excited to fire once at short latency (7.7 ± 1.9 ms) by ipsilateral trunk skin stimulation (Fig. 7 and Table 1). However, the rdlcs were also excited to fire, and at even shorter latency (6.5 ± 1.7 ms), by head‐skin stimuli. If the stimulus threshold to initiate swimming is 100%, the threshold was 95 ± 3% for rdlc EPSPs and 97 ± 3% for rdlc spiking. The large EPSPs in rdlcs following head‐skin stimulation were similar to those evoked by trunk stimulation (analysed below spike threshold) (Table 1). Basic cellular properties were measured for 14 rdlcs (Table 1). When depolarizing current was injected, rdlcs fired a single action potential at threshold but, at higher current levels, they fired repetitively (Fig. 7 *E*). Neurobiotin fillings showed that they have dorsolaterally located, uni‐ or multipolar somata (diameter 13–16 μm) with several short dendrites, some of which are on the initial part of the axon (Fig. 7 *F*). Their single axon runs ventrally to cross the mid‐line at a similar longitudinal level as the soma and, on the contralateral side, it bifurcates to ascend into the hindbrain and descend into the spinal cord.

**Table 1:** **Measurements of rdlc EPSPs and spikes in response to ipsilateral head and trunk skin stimulation and basic properties**

Parameter	Head stimulation	Trunk stimulation
EPSP latency	4.9 ± 1.3 ms (20)	5.8 ± 1.6 ms (15)
EPSP amplitude (no spike)	11.6 ± 5.1 mV (10)	14.6 ± 8.6 mV (8)
EPSP 10–90% rise time	2.0 ± 1.7 ms (10)	1.4 ± 0.3 ms (8)
EPSP time to peak	8.1 ± 1.7 ms (10)	7.7 ± 1.3 ms (8)
EPSP duration at 50% amplitude	5.7 ± 1.5 ms (10)	5.7 ± 1.9 ms (8)
Spike latency	6.5 ± 1.7 ms (20)	7.7 ± 1.9 ms (14)
Input resistance	464 ± 208 MΩ (14)	
Capacitance	14.5 ± 4.0 pF (14)	
Resting membrane potential	−51.0 ± 4.7 mV (14)	
Spike threshold	−18.2 ± 5.9 mV (14)	
Spike peak amplitude	22.1 ± 7.8 mV (14)	
Spike width at half peak	0.65 ± 0.20 ms (14)	

For each parameter, the mean ± SD and number of measured neurons (in parenthesis) are given.

The anatomical and physiological properties of rdlcs and their responses to trunk‐skin stimulation correspond to those of spinal dlcs (Clarke and Roberts, 1984; Roberts and Sillar, 1990; Li *et al*. 2003). The critical additional feature is that rdlcs are also excited following head‐skin stimulation (Fig. 7 *C* and *D*). This excitation is at slightly shorter latencies than that from trunk‐skin stimulation where conduction times from a more distant stimulus location would be greater. The EPSP latencies in rdlcs following head‐skin stimulation were 4.9 ± 1.3 ms, which is similar to latency ranges for EPSPs in spinal cord sensory interneurons following trunk‐skin stimulation (dlc 5.0–7.0 ms; dla 3.5–6.7 ms), where, by paired recording, their connections from the Rohon‐Beard sensory neurons were shown to be monosynaptic (Roberts and Sillar, 1990: Li *et al*. 2003, 2004). It is also within the range of the 4.4 ± 0.5 ms latencies measured in tINs lying more rostral, and therefore closer to the stimulus, which were also presumed to be monosynaptic (Table 1) (Buhl *et al*. 2012). This suggests that rdlc EPSPs result from direct synapses made by the descending axons of primary trigeminal sensory neurons innervating the head skin.

The connections of rdlcs onto dINs were investigated by making paired recordings from them with the excitatory dINs on the opposite, unstimulated side. In 11 paired recordings, neurobiotin filling showed that the rdlc axon crossed ventrally to lie in close proximity to the recorded dIN dendrites. To stimuli that evoked swimming, the rdlcs fired only once and EPSPs were recorded in dINs. However, these EPSPs had long and variable latencies (Fig. 7 *F* and *G*) and their shapes suggest that they were the compound sum of presynaptic spikes occurring at different delays. The delays from rdlc spikes to the onset of dIN EPSPs ranged from 2.9 to 7.1 ms (mean ± SD: 4.5 ± 0.9 ms, *n* = 27 initiations in three pairs) (Fig. 7 *G*). These delays are much longer than seen in other monosynaptic, tadpole synapses (Li *et al*. 2003; Buhl *et al*. 2012). Furthermore, when an rdlc spiked in response to current injection, no postsynaptic potentials were seen in the recorded dIN. These observations suggest that additional neurons lie interposed in the pathway between rdlcs and dINs on the opposite side, and that this contributes to the delay in the response on the unstimulated side. Although we cannot rule out the possible contribution of other neurons, our results suggest that the rdlcs are strong candidates for bringing the excitation to the contralateral side following head‐skin stimulation, acting via some currently unidentified interposed neurons.

## Discussion

Defining complete, neuron by neuron pathways, from sensory stimulation to the initiation of co‐ordinated movements, is difficult (Vinay *et al*. 1995; Sparks, 2002; Viana Di Prisco *et al*. 2005), even in invertebrates (Esch *et al*. 2002). To trace the origins of the vertebrate networks initiating locomotion, we chose a very simple case: swimming of hatchling *Xenopus* tadpoles initiated by touching one side of the head. Video recordings showed that tadpoles first make a strong flexion whose direction, similar to the subsequent swimming path, is variable and unpredictable (Fig. 1 *A* and *B*) (Boothby and Roberts, 1995). Similar fictive responses, recorded in immobilized tadpoles (Fig. 1 *C–E*), show the underlying motor activity. We previously defined a sensory pathway for tSt receptors to initiate swimming on the stimulated side following head touch (Buhl *et al*. 2012). In the present study, we investigated how tSt firing on one side also leads to dIN excitation on the unstimulated side, as is necessary for bilateral initiation. Whole‐cell recordings show that tSts directly excite rostral dorsolateral commissural neurons (rdlcs) to fire once. These neurons are the most rostral members of a population of spinal sensory pathway neurons excited by trunk skin afferents and carrying excitation to the opposite side (Clarke and Roberts, 1984; Roberts and Sillar, 1990; Li *et al*. 2003). Perhaps surprisingly, paired recordings from rdlcs and dINs on opposite sides failed to show direct connections and also suggested that the delays between rdlc firing and dIN EPSPs following skin stimulation were too long for direct connections. We conclude that, instead of directly exciting dINs, rdlcs do so indirectly via some unidentified neuron type. This step introduces a delay within the initiation process and also the potential for flexibility.

We now consider the main questions that we have addressed. What is required for the tadpole to decide to initiate locomotion? How does it organize alternation when swimming starts at the same time as avoiding synchronous activation of antagonist muscles? We will also ask what makes the swimming direction unpredictable. Similar to other vertebrates (lamprey: Viana Di Prisco *et al*. 2005; cat: Aoki and Mori, 1981; rat: Vinay *et al*. 1995), tadpole locomotion can be initiated by head‐skin stimulation. The initiation decision requires a series of thresholds to be crossed, ultimately leading to co‐ordinated firing in the bilateral populations of reticulospinal dINs.

### Decision‐making steps for initiation of locomotion

The sensitivity of the touch‐sensory nerve endings will determine the first threshold when one sensory neuron fires once. Increasing the stimulus strength will recruit more sensory neurons, each firing a single spike (Buhl *et al*. 2012), however evidence on receptive field overlap suggests that the maximum number will be less than 10 (Roberts, 1980). Sensory neuron spikes then bring sensory pathway neurons with ipsilateral (tINs) and commissural (rdlcs) axon projections to threshold. This step in the pathway can amplify the excitation from a few sensory neurons by the recruitment of more sensory pathway neurons. In the case of tINs, there is further amplification because they can fire multiply (Buhl *et al*. 2012). The limited number of sensory pathway neurons may also help to restrict the maximum possible excitation if large numbers of head‐skin sensory neurons are excited simultaneously. Finally, the sensory pathway neurons excite the reticulospinal dIN neurons, the critical neurons in the generation of swimming, either directly (tINs) or indirectly (rdlcs) via unknown neurons.

Our evidence suggests that the decision to swim takes place in the populations of reticulospinal dIN neurons that synaptically drive the other swim neurons, including motoneurons, to fire on each cycle (Roberts *et al*. 2010; Li, 2011). Our new evidence indicates that, following skin stimulation, excitation builds up in dINs until their firing threshold is crossed on one side. Because they are electrically coupled to each other, recruitment of firing in a few dINs leads to recruitment of the whole dIN population on this side and, as a result, the initiation of swimming. As this happens, excitation will also be building up on the other side. Our whole‐cell recordings of responses in single or pairs of dINs to head‐skin stimulation show that their first firing almost always precedes the start of swimming recorded in the ventral roots (95.4%: 209 of 219 trials). This confirms the results from our previous study of dINs on the stimulated side where dIN firing also preceded ventral root swimming (98%: 170 of 173 trials; Buhl *et al*. 2012) supporting our view that dIN population firing is the necessary precursor to swimming and the key decision‐making step in initiation. Modelling of the hindbrain dIN population has shown that, without electrical coupling, recruitment is gradual and often incomplete, whereas, with electrical coupling, recruitment follows a step function: where dINs are recruited all‐or‐none (Hull *et al*. 2015). This is exactly what is needed for a tadpole to decide to swim.

*Xenopus* reticulospinal dINs are probable homologues of *Chx10*‐expressing V2a neurons in larval zebrafish (Kimura *et al*. 2013), which have similar distribution, anatomy and functions during swimming. The optogenetic activation of V2a neurons in the hindbrain using channelrhodopsin evokes swimming, whereas their inactivation using archaerhodopsin3 or halorhodopsin reliably stops on‐going swimming. Taken together with our evidence, this points to these hindbrain neurons being the place where, together with electrical coupling, a simple threshold mechanism is the basis for the decision to swim. Because both sides are depolarized by the time that dINs on one side fire, inhibition from the side starting first could allow rebound firing on the opposite side and help ensure that the response initiated is bilateral (Moult *et al*. 2013).

### Asymmetric initiation of motor activity

When animals make even simple motor responses, they need to be co‐ordinated from the start and, in mammals, this may be one reason why reaction times are long and variable, even for simple eye movements (90–400 ms; Gold and Shadlen, 2007). In the tadpole, reaction times to the start of swimming following skin stimulation are also long and variable (15–87 ms) compared to reflexes (∼10 ms; Li *et al*. 2003). Perhaps our most striking observation is the very organized firing patterns of reticulospinal dIN neurons on each side of the body, which ensures alternation of body contractions from the start (Figs 2 and 3). This firing pattern appears to depend primarily on an asymmetric underlying excitatory drive from the head‐skin sensory pathways to each side of the body. Because reticulospinal dINs are known to excite reciprocal inhibitory commissural neurons (Li *et al*. 2006), we would expect to see IPSPs on one side when dINs are recruited on the other side. Such IPSPs were not apparent (Figs 2 and 3). This may be because inhibitory commissural interneurons are not yet firing or that the dIN membrane potential is very close to the IPSP reversal potential (Sautois *et al*. 2007). Whether there is some additional contribution from reciprocal inhibition remains to be examined.

How is sensory excitation organized to produce asymmetrical input to the motor system? The clearest evidence is that tINs directly excite ipsilateral dINs via glutamatergic synapses to produce short‐latency EPSPs. These EPSPs can sum to threshold and lead to recruitment of dIN firing at short latencies of ∼15 ms (Fig. 2 *C*) (Buhl *et al*. 2012). Swimming then starts on the stimulated side. Regardless of whether the dINs on the stimulated side fire, the next dIN population to be recruited is on the unstimulated side. The excitatory input here is variable and ‘bumpy’ (Fig. 4) but builds up to threshold so that firing occurs at ∼35 ms. Finally, after the unstimulated side has fired, excitation on the stimulated side builds up in a very variable fashion and dINs fire at ∼60 ms. The asymmetrical pattern of sensory pathway excitation to the reticulospinal dINs therefore co‐ordinates a rather variable but strictly alternating response to stimulation.

**Figure 8:**
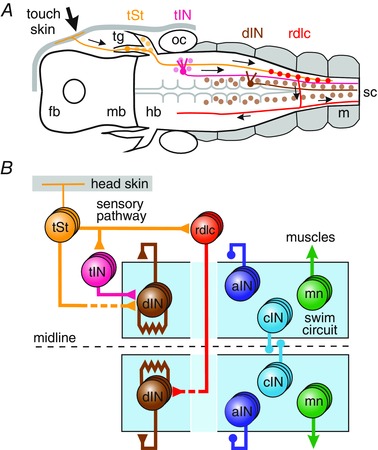
**Swim initiating network** *A*, brain in dorsal view and the head‐skin trigeminal pathways initiating swimming on the same or opposite sides. Dots show locations of the neuron populations, not their real numbers: touch‐sensory trigeminal neurons (tSt, yellow), sensory pathway neurons (tIN, pale magenta; rdlc, red), electrically coupled reticulospinal excitatory neurons (dIN, brown). Fb, forebrain; hb, hindbrain; mb, midbrain; m, muscles; oc, otic capsule; sc, spinal cord; tg, trigeminal ganglion. *B*, functional diagram of the network including the central pattern generator (swim circuit) neurons: inhibitory interneurons (cIN, blue; aIN, purple) and motoneurons (mn, green). Continuous lines indicate evidence for a monosynaptic connection. Dashed lines indicate indirect connections (delay). Large circles represent populations of neurons. Small circles (inhibitory) and triangles (excitatory) are synapses and, when they contact a box, they connect to all neurons in the box.

Our present understanding of the pathways exciting reticulospinal neurons following skin stimulation is summarized in Fig. 8. At present, we can account for the early excitation by tINs on the stimulated side but the neurons producing the more slowly increasing excitation on each side remain unknown. These neurons must fire repetitively following excitation from the head sensory pathway to provide long‐lasting AMPA receptor and NMDA receptor activation of dINs through EPSPs which could sum to bring dINs to their firing threshold after the observed variable delays. Their sensory excitation on the stimulated side could be direct from trigeminal sensory axons or via tINs. On the unstimulated side, we expect these neurons to be excited by commissural sensory pathway neurons (rdlcs) and to be excited more strongly than on the stimulated side. This would produce the required asymmetry of dIN activation. It is interesting that, in other motor systems, a group of ‘trigger’ neurons is often present. These are excited by sensory input and activate rhythm‐generating neurons that are the analogues of reticulospinal dINs (Brodfuehrer and Friesen, 1986).

### Unpredictable direction of response

The decision to respond appropriately to sensory stimulation is central to an animal's survival. Swimming away from danger is one of the few defence mechanisms for hatchling *Xenopus* tadpoles. This allows them to avoid predators such as adult *Xenopus* and water scorpions, as well as the larvae of beetles, damselflies and dragonflies (Lawler, 1989). To avoid danger, a widespread strategy among animals is to move away in an unpredictable direction (Domenici *et al*. 2011). The ballistic escape responses of invertebrates or fish are fast but predictable (crayfish: Olson and Krasne, 1981; fish: Korn and Faber, 2005), and this can be exploited by predators such as water snakes that learn which way prey will turn (Catania, 2009). We have found, in the tadpole, that the side contracting first is unpredictable for stimuli close to threshold. In addition, regardless of the side of the first bend, the direction of subsequent swimming is very variable (Boothby and Roberts, 1995) (Fig. 1 *B*). When stronger stimuli are given to the head skin, the latency to dIN firing is shorter and they reliably fire first on the stimulated side. The response becomes a fast but predictable flexion to the stimulated side and our evidence from lesioning suggests that this response depends on tIN excitation to dINs (Fig. 5). Stronger stimuli recruit more tINs to fire more impulses (Buhl *et al*. 2012). Our hypothesis is that the unpredictable response to weaker stimuli depends on the unreliable firing of the sensory pathway tIN neurons. Weaker stimuli will recruit fewer tINs to fire fewer impulses and make ipsilateral dIN firing, and therefore swimming direction, unreliable. Furthermore, the tINs have another property that could enhance unpredictability. Paired recordings show that their synapses onto dINs fail in 50% of trials (Buhl *et al*. 2012). By contrast, the commissural pathway exciting dINs on the unstimulated side leads to a slower and variable build‐up of excitation, that leads to reliable firing within a defined time window. The result is that swimming starts later on the unstimulated side when excitation within the stimulated side fails to recruit dINs.

### Conclusions

The task faced by the tadpole as it initiates swimming is essentially the same as that for many other directed motor responses. The decision to swim involves key neurons, in this case excitatory rerticulospinal neurons, crossing the firing threshold as a result of incoming signals, in this case from head‐skin sensory pathways. Even in the very simple tadpole motor system, reaction times for the initiation of swimming are long compared to reflexes. This appears to be a common feature of other decision‐making processes (Gold and Shadlen, 2007). Successful implementation of the decision again involves dealing with a common requirement in motor systems: antagonists must both be activated but synchronous co‐activation must be avoided. In the tadpole, we have shown how this is built into the decision‐making process by an asymmetry in the pattern of excitation to the reticulospinal neurons. A further consequence of this asymmetry is that, although the response is largely stereotyped for stronger stimuli, the direction of response following weaker stimuli is unpredictable. In the tadpole, this unpredictability may aid survival; in other systems, mechanisms may be needed to control or prevent it.

## Additional information

### Competing interests

The authors declare that they have no competing interests.

### Author contribution

The experiments were performed in the neurobiology laboratory at the University of Bristol. All of the authors contributed to conceiving, designing and carrying out the experiments, as well as the analysis of data and writing of the manuscript. All authors approved the final version of the manuscript submitted for publication.

### Funding

This work was supported by the BBSRC grant (BB/G006652/1).
